# Severe ARDS caused by *Strongyloides stercoralis* hyperinfection in a patient with Sjögren’s syndrome: a case report

**DOI:** 10.3389/fmed.2026.1892861

**Published:** 2026-08-10

**Authors:** Ting Lyu, Song Gao, Wenjun Wang, Wei Liu

**Affiliations:** Department of Critical Care Medicine, Xishan People’s Hospital of Wuxi City, Wuxi, China

**Keywords:** acute respiratory distress syndrome, eosinopenia, glucocorticoid, MetaCAP, Sjögren’s syndrome, *Strongyloides stercoralis*

## Abstract

**Background:**

*Strongyloides stercoralis* (*S. stercoralis*) is a neglected tropical disease that can be fatal in immunocompromised hosts. Diagnosis is challenging due to nonspecific clinical manifestations and low sensitivity of conventional stool examination. Patients with autoimmune diseases receiving long-term glucocorticoid therapy are at high risk of hyperinfection syndrome, which can rapidly progress to acute respiratory distress syndrome (ARDS).

**Case presentation:**

A 65-year-old man with an 8-year history of Sjögren’s syndrome and chronic interstitial lung disease, who had been on long-term oral methylprednisolone, tripterygium glycosides, and hydroxychloroquine, presented with abdominal pain and vomiting. He rapidly developed severe ARDS requiring invasive mechanical ventilation. Laboratory tests showed persistent eosinopenia (0.01 × 10^9^/L) and lymphopenia. Bronchoalveolar lavage fluid was subjected to metagenomic capture sequencing (MetaCAP), which revealed *S. stercoralis* [16,030 reads per million (RPM)] and cytomegalovirus (14,397RPM). Sputum smear microscopy showed motile *S. stercoralis* larvae, confirming the diagnosis. The patient was treated with albendazole (0.4 g via nasogastric tube twice daily) combined with ivermectin (12 mg via nasogastric tube once daily) for strongyloidiasis, together with ganciclovir for cytomegalovirus. Because of severe ARDS and possible autoimmune flare, methylprednisolone (80 mg intravenously every 12 h followed by tapering) was cautiously administered under effective anti-infective coverage. Two days after treatment, the oxygenation index improved from 77 mmHg to 214 mmHg. One week later, repeat MetaCAP showed a marked reduction of *S. stercoralis* reads to 69RPM and cytomegalovirus 9 RPM. The patient was successfully extubated and discharged after consolidation therapy. At nine-month follow-up he remained well.

**Conclusion:**

This case demonstrates that *S. stercoralis* hyperinfection can occur without eosinophilia in immunocompromised patients with autoimmune diseases and can rapidly progress to ARDS. MetaCAP of bronchoalveolar lavage fluid enables rapid and sensitive diagnosis. Short-term glucocorticoid therapy to control ARDS and autoimmune disease activity is feasible and safe provided that effective anti-infective treatment is in place.

## 1 Introduction

*Strongyloides stercoralis* (*S. stercoralis*) is a facultative soil-transmitted helminth. Its filariform larvae invade through skin or mucosa, disseminate hematogenously, and cause persistent intestinal infection that may become life-threatening (mortality 30–70%) if untreated (1). The disease is endemic in tropical, subtropical, and temperate regions with poor sanitation (2), but has become rare in China (3).

Diagnosis is challenging: clinical manifestations are nonspecific, and a single stool examination detects fewer than 30% of cases. Patients may also lack peripheral eosinophilia—the most familiar warning sign of helminth infection—leading to frequent misdiagnosis (4). The risk is particularly high in patients on long-term immunosuppressive therapy (e.g., glucocorticoids, immunomodulators, or chemotherapy), who are more prone to severe disease (5). In this setting, clinicians often face a difficult trade-off between controlling the underlying autoimmune disease and managing infection, especially when glucocorticoids are involved.

We report a case of *S. stercoralis* hyperinfection in a patient with Sjögren’s syndrome receiving long-term immunosuppression, who presented with non-specific gastrointestinal symptoms and rapidly developed severe ARDS. The pathogen was promptly identified by metagenomic capture sequencing (MetaCAP) of bronchoalveolar lavage fluid, and the patient was successfully treated with antiparasitic agents and short-course glucocorticoids. This case underscores three key messages: a. strongyloidiasis should be suspected in immunocompromised patients with unexplained respiratory failure, even without eosinophilia; b. MetaCAP enables rapid, accurate diagnosis; c. short-term glucocorticoid use is feasible and safe when anti-infective coverage is adequate.

## 2 Case presentation

### 2.1 General information

A 65-year-old man was admitted to the Department of Gastroenterology on August 14, 2025, with complaints of “abdominal pain accompanied by nausea and vomiting of retained food for 3 days.” His past medical history included Sjögren’s syndrome for 8 years, for which he had been receiving long-term oral therapy with tripterygium glycosides (10 mg orally twice daily), hydroxychloroquine (0.2 g orally twice daily), and methylprednisolone (4 mg orally twice daily); and chronic interstitial pulmonary fibrosis for 8 years, for which he took pirfenidone (0.4 g orally three times daily), The patient had been generally well, without recurrent dyspnea or wheezing indicating worsening of pulmonary fibrosis, and no frequent hospitalizations. On July 26, 2025, he developed fever and was admitted to a tertiary hospital. Sputum smear for acid-fast bacilli was negative, but sputum culture revealed Aspergillus flavus. He responded to intravenous voriconazole and continued on oral voriconazole until the current admission, during which he remained well; however, clearance of Aspergillus flavus was not confirmed by follow-up sputum culture. He also had a 10-year history of hypertension (treated with oral amlodipine besylate and sacubitril/valsartan) and a 8-year history of atrial fibrillation for which he took rivaroxaban (10 mg orally once daily)and digoxin (0.125 mg orally once daily). There was no significant family history, no history of travel to endemic areas, no occupational or daily activities involving soil exposure, and no known close contact with infected individuals and animals.

### 2.2 Present illness

Three days before admission, the patient developed persistent upper abdominal pain after a large meal, accompanied by nausea and vomiting of retained food on several occasions (no coffee-ground material was observed). He had passage of flatus and a small amount of stool. There was no referred pain to the lower back, no migratory right lower quadrant pain, and no fever. He then presented to our emergency department.

Laboratory investigations revealed: white blood cell count 14.64 × 10^9^/L, neutrophils 12.66 × 10^9^/L (86.5%), lymphocytes 7.7%, hemoglobin 125 g/L, and C-reactive protein (CRP) 16.3 mg/L. Arterial blood gas analysis showed lactate 3.66 mmol/L. D-dimer was 0.52 μg/mL. Contrast-enhanced abdominal CT demonstrated: (1) portal venous gas; (2) bowel wall gas in the horizontal part of the duodenum and proximal jejunum, with localized wall thickening and luminal dilation.

In the emergency department, he received ceftriaxone, anisodamine, omeprazole and intravenous fluids. He was treated with ceftriaxone (2 g intravenous drip (ivdrip) qd), anisodamine (10 mg ivdrip qd), omeprazole (40 mg ivdrip qd), and intravenous fluids in the emergency department. His abdominal pain slightly improved and the frequency of vomiting decreased. He was admitted to the Department of Gastroenterology with a diagnosis of “ileus.” After admission, He received fasting, parenteral nutrition, and intravenous imipenem/cilastatin (1.0 g q12h) for anti-infective therapy.

Three days later, his abdominal pain resolved and flatus returned; however, he developed cough, sputum production, hemoptysis and significant wheezing, without fever, chills or chest pain. Because of respiratory failure, the patient was transferred to the Intensive Care Unit (ICU).

### 2.3 ICU physical examination

Vital signs: afebrile, blood pressure was 132/62 mmHg, heart rate was 143 beats per minute with irregular rhythm (atrial fibrillation), respiratory rate was approximately 45 breaths per minute, and peripheral oxygen saturation (SpO_2_) was 88% on high-flow oxygen therapy (fraction of inspired oxygen 70%, flow rate 50 L/min).

Breath sounds were coarse bilaterally, with a few moist rales. No pathological murmurs were audible. The abdominal wall was slightly tense, with tenderness in the periumbilical and right mid-abdominal regions, and mild rebound tenderness was present. Muscle strength was normal (grade 5/5) in all four limbs. No skin rash or petechiae were observed. The patient was alert, oriented and conversational. Neck was supple, with no nuchal rigidity, and muscle strength was normal in all extremities.

### 2.4 Auxiliary examinations

On August 18, 2025, arterial blood gas analysis (FiO_2_ 70%) showed: pH 7.430, PaCO_2_ 30.6 mmHg, PaO_2_ 54 mmHg, SaO_2_ 89.5%, standard bicarbonate 21.5 mmol/L, actual bicarbonate 19.9 mmol/L, base excess −3.6 mmol/L, alveolar-arterial oxygen gradient 411.5 mmHg, lactate <1.0 mmol/L; the oxygenation index (PaO_2_/FiO_2_) was 77 mmHg.

**Figure 1:**
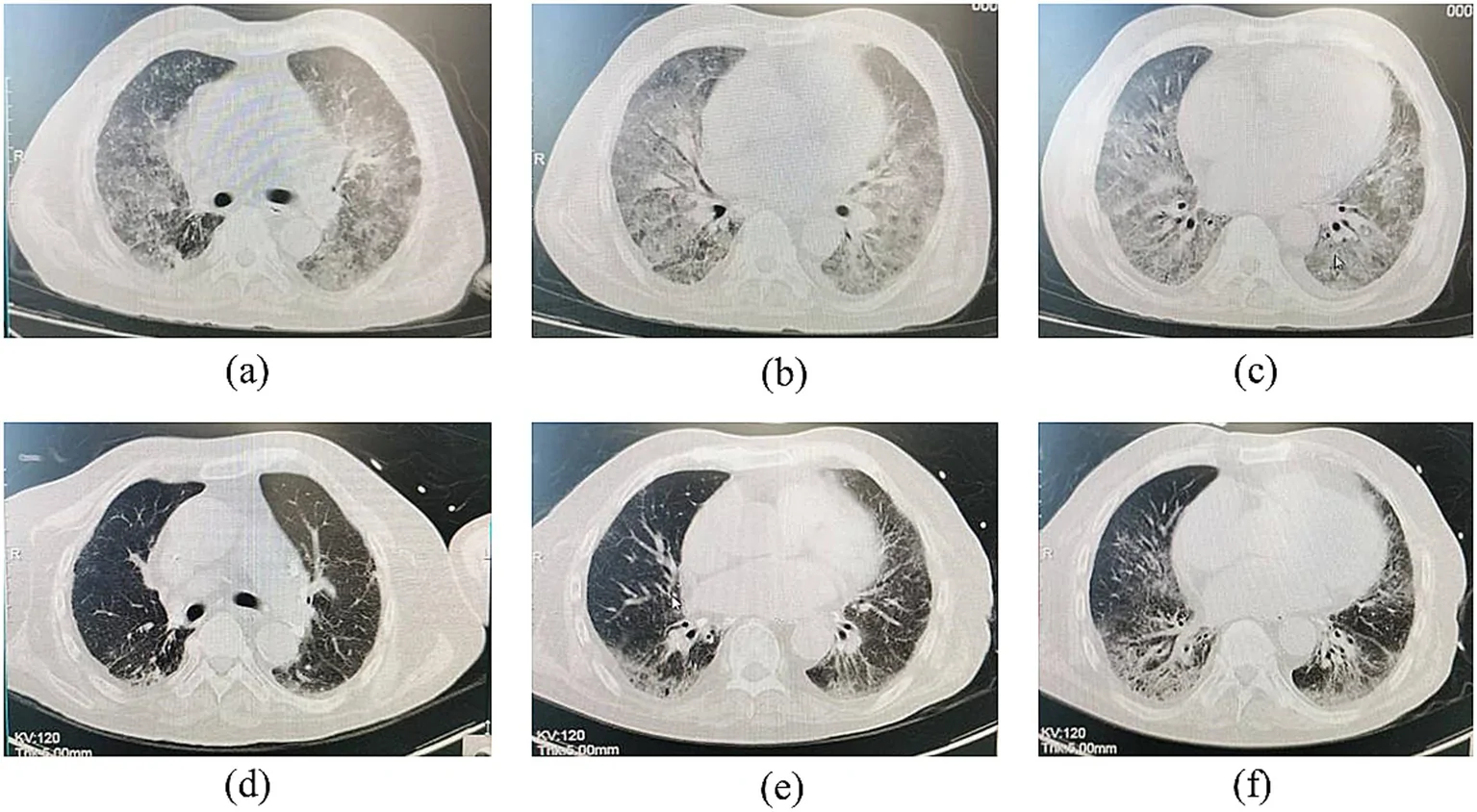
Chest CT (**a–c**: before treatment, diffuse exudative opacities in both lungs; **d–f**: after 10 days of treatment, marked resolution of exudative opacities).

Blood routine with CRP: white blood cell count 7.88 × 10^9^/L, neutrophils 91.7% (absolute count 7.22 × 10^9^/L), eosinophils 0.1% (absolute count 0.01 × 10^9^/L), lymphocytes 5.6%, hemoglobin 92 g/L, CRP 143.1 mg/L. Routine stool microscopy revealed no obvious abnormalities, with no larvae or ova identified. Procalcitonin was 0.10 ng/mL. Lymphocyte subsets: total T cells 496/μL (reference 902–2,665), CD4^+^ T cells 289/μL (reference 453–1,365), CD8^+^ T cells 182/μL (reference 281–1,106), B cells 32/μL (reference 155–290), NK cells 261/μL (reference 150–1,000), total lymphocytes 825/μL (reference 1,530–3,700), CD4^+^/CD8^+^ ratio 1.6 (reference 0.6–2.9). Infectious disease markers (hepatitis B, hepatitis C, HIV, syphilis) were all negative. Biochemistry: total bilirubin 26.90 μmol/L, direct bilirubin 14.5 μmol/L, alanine aminotransferase 28 U/L, aspartate aminotransferase 33 U/L, lactate dehydrogenase 515 U/L, prealbumin 86.5 mg/L, albumin 28.6 g/L, blood urea nitrogen 3.12 mmol/L, creatinine 33.8 μmol/L, creatine kinase isoenzyme 21 U/L, creatine kinase 73 U/L (trends of inflammatory markers and blood gas parameters are shown in Table 1). Chest CT revealed diffuse exudative opacities in both lungs (Figures 1a–c).

**Table 1:** Trends of inflammatory markers, eosinophil count and blood gas parameters.

Date	Test item						
	White blood cell (*10^9/L) (3.5–9.5)	Eosinophil percentage (%) (0.4–8)	Absolute eosinophil count (*10^9/L) (0.02–0.52)	Procalcitonin (ng/ml) (0.0–0.25)	CRP (mg/L) (0.0–10)	Oxygenation index (mmHg)	PaCO_2_ (mmHg)
8.17	5.43	1.3	0.07	0.1	<0.5	100	28
8.18	7.88	0.1	0.01	0.37	143.1	77	30.6
8.19	10.26	2.3	0.24	0.41	144.7	118	35.7
8.20	8.02	0.1	0.01	1.0	234.2	214	53.7
8.21	12.37	0.0	0.00	0.74	150.2	220	52.3
8.22	10.66	0.0	0.0	0.0	68.5	322	43.7
8.25	11.0	0.0	0.0	0.0	36.7	325	44.0
8.30	13.35	0.0	0.0	0.13	15.3	422	29.9
9.02	14.86	0.0	0.0	0.07	1.9	348	28.2

CRP, C-reactive protein; PaCO_2_, partial pressure of carbon dioxide.

The patient rapidly developed respiratory failure. Based on the blood gas analysis and chest CT findings, ARDS was diagnosed. Given the patient’s history of autoimmune disease, the etiology of ARDS was considered to be either interstitial pneumonia due to autoimmune activity or overwhelming infection secondary to immunosuppression.

**Figure 2:**
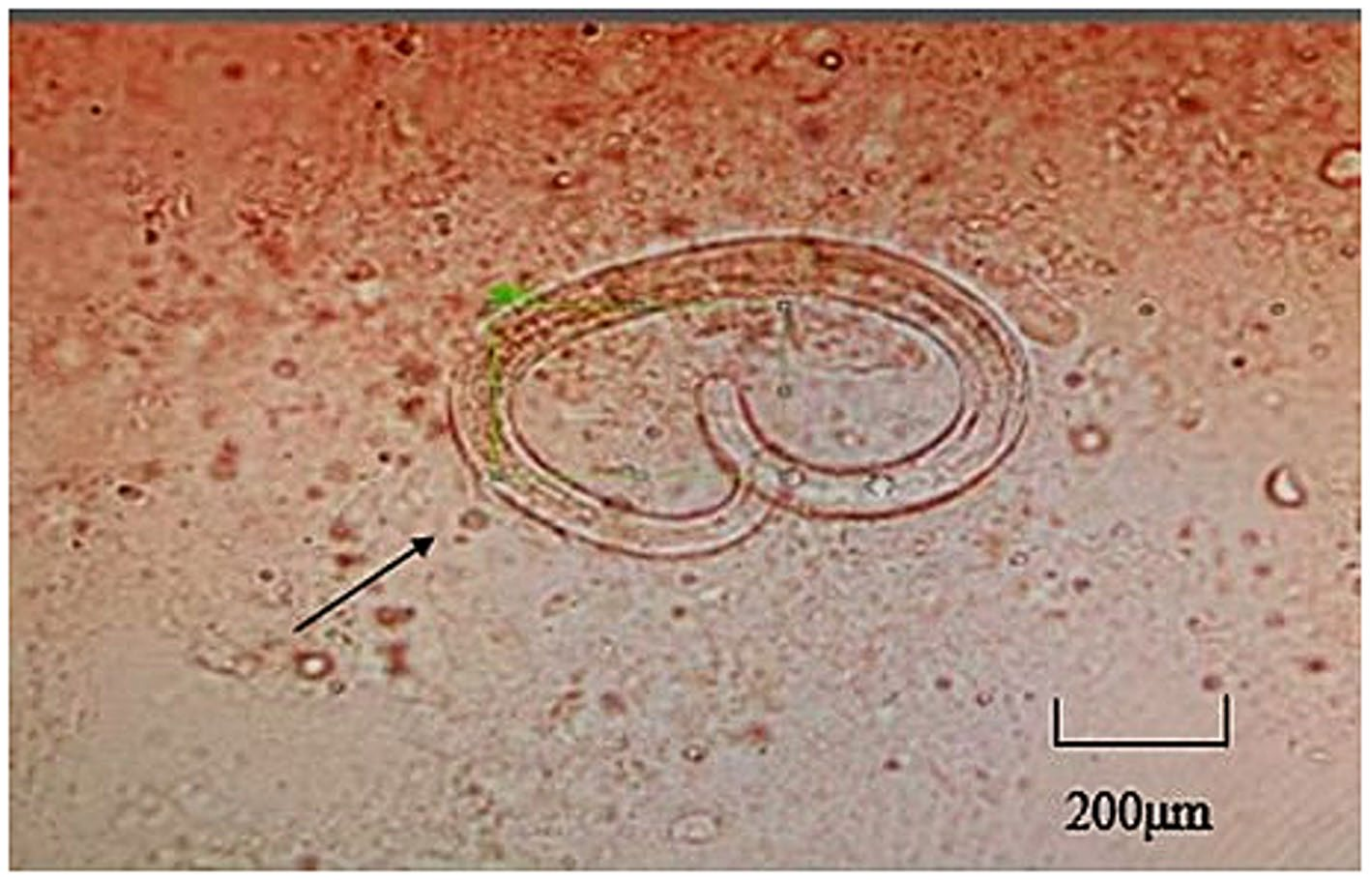
Microscopic examination of sputum showing *S. stercoralis* larvae (×400).

To quickly identify any pathogenic infection, fiberoptic bronchoscopy was performed on the second day after ICU admission, and bronchoalveolar lavage fluid (BALF) was collected for metagenomic capture sequencing (MetaCAP). Bronchoscopy revealed large amounts of bloody secretions and scattered hemorrhagic spots on the airway mucosa. Twenty-four hours later, MetaCAP results showed 16,030 reads per million (RPM) for *S. stercoralis* and 14,397 RPM for cytomegalovirus (CMV) (Table 2). All other tested pathogens, including bacteria, fungi, mycobacteria, mycoplasma, chlamydia, rickettsiae, and spirochetes, were negative. Because MetaCAP suggested a parasitic infection, we repeated bronchoscopy and performed sputum smear microscopy, which revealed motile *S. stercoralis* larvae (Figure 2).

**Table 2:** BALF MetaCAP results.

Time	Genus				Species		
	Type	Name	Reads	Relative abundance	Name	Reads	Confidence
Before treatment	dsDNA	Cytomegalo virus	14,397	98.67%	Cytomegalo virus	14,397	99%
	Parasites	Strongyloides	16,114	99.98%	*S. stercoralis*	16,030	99%
After treatment	dsDNA	Cytomegalo virus	9	7.13%	Cytomegalo virus	9	99%
	Parasites	Strongyloides	170	46.01%	*S. stercoralis*	69	99%
	Gram-negative bacteria	Stenotrophomonas	126,029	86.51%	*Stenotrophomonas maltophilia*	111,983	99%

Reads: the number of high-quality reads that can be aligned to the pathogen after normalizing the total sequencing reads to one million (RPM).

### 2.5 Treatment and outcome

After transfer to the ICU, the patient received orotracheal intubation with mechanical ventilation. Empirical anti-bacterial therapy was initiated with imipenem/cilastatin (1.0 g ivdrip q12h), and anti-fungal therapy with voriconazole (0.2 g ivdrip q12h).

Based on the BALF MetaCAP results, anthelmintic therapy was given with albendazole (0.4 g via nasogastric tube twice daily) combined with ivermectin (12 mg via nasogastric tube once daily). Antiviral therapy with ganciclovir (0.25 g ivdrip q12h) was also administered.

Repeated fiberoptic bronchoscopy was performed for sputum aspiration to promote drainage, and prone positioning was used to improve lung function.

Considering the patient’s autoimmune disease and long-term glucocorticoid use leading to immunocompromise, human immunoglobulin (10 g ivdrip qd for 7 days) and thymalfasin (1.6 mg subcutaneous qd for 10 days) were given to enhance immunity.

**Figure 3:**
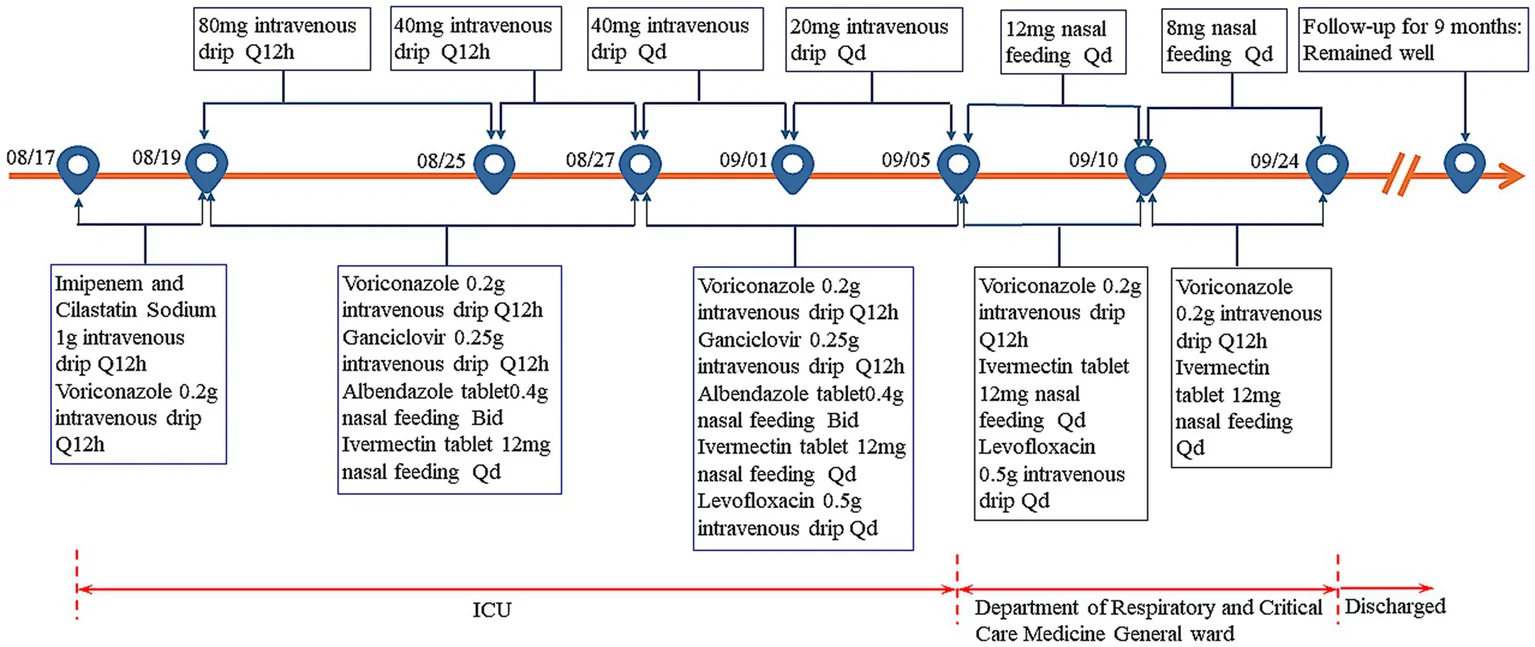
Methylprednisolone regimen (above the line) and anti-infective regimen during the clinical course (below the line).

Because the patient’s ARDS could not rule out an excessive inflammatory response triggered by infection, methylprednisolone (80 mg ivdrip q12h) was administered, with gradual tapering down to 12 mg via nasogastric tube once daily. Concurrently, pirfenidone (0.4 g via nasogastric tube three times daily) was continued to manage pulmonary fibrosis. For Sjögren’s syndrome, baseline medications were continued: tripterygium glycosides (10 mg via nasogastric tube twice daily) and hydroxychloroquine sulfate (0.2 g via nasogastric tube twice daily). Additional supportive care included rate control for atrial fibrillation and nutritional support. The treatment process is shown in Figure 3.

After 2 days of treatment, the patient’s respiratory function improved significantly, with the oxygenation index rising from 77 mmHg to 214 mmHg. One week later, repeat BALF MetaCAP showed a marked reduction in *S. stercoralis* reads 69 RPM and CMV 9 RPM (Table 2), while also identifying *Stenotrophomonas maltophilia* (111,983RPM), which was confirmed by concurrent sputum culture; levofloxacin was accordingly added based on susceptibility testing.

Ten days later, the endotracheal tube was successfully removed, and repeat chest CT showed significant resolution of bilateral exudative opacities (Figures 1d–f). After about 2 weeks of treatment, the patient’s condition had markedly improved, and he was transferred to the Department of Respiratory and Critical Care Medicine (PCCM)for further consolidation therapy for about 20 days, and was then discharged without ongoing oral antiparasitic therapy, while his regular medications for Sjögren’s syndrome, interstitial pulmonary fibrosis, and other comorbidities were continued. At 9 months of follow-up, he remained in his usual state of health without any specific complaints.

## 3 Discussion

### 3.1 Epidemiology and host risk factors

*S. stercoralis* is estimated to infect as many as 613.9 million people worldwide, and seropositivity rates as high as 11–14% have been reported in some southern regions of China (1–3). Most infected individuals are asymptomatic, but once the host becomes immunocompromised, a fatal hyperinfection syndrome can occur (4, 5). Glucocorticoids are the most common trigger – even short-term, low-dose use can disrupt the host–parasite immune balance (6). The patient had been receiving long-term oral glucocorticoids (methylprednisolone) and tripterygium glycosides, both immunosuppressive agents, and his lymphocyte subset analysis further confirmed immunocompromised status, placing him in a typical high-risk group.

### 3.2 Clinical features

Both larvae and adult worms of *S. stercoralis* are pathogenic. Adult females in the gut cause local inflammation (fever, nausea, vomiting, diarrhea, abdominal pain); after oviposition, filariform larvae migrate to the lungs, causing hemorrhage and inflammation manifesting as cough, hemoptysis, dyspnea, and wheezing. Severity depends on host immunity and parasite burden: immunocompetent hosts are often asymptomatic, whereas immunocompromised patients may develop disseminated infection involving the brain, lungs, heart, liver, kidneys, and endocrine glands, potentially leading to multiorgan failure (7). Transient skin lesions (urticaria, larva currens) may also occur (8). Our patient presented with abdominal distension, pain, nausea, and vomiting, followed 2 days later by respiratory symptoms, without skin involvement. The clinical features were consistent with strongyloidiasis.

### 3.3 Diagnostic pitfall: the meaning of normal eosinophil counts

Eosinophilia is a hallmark of many parasitic infections. However, in *S. stercoralis* hyperinfection, due to glucocorticoid-induced Th2 cell apoptosis, reduced eosinophil production and inhibition of mast cell responses, peripheral eosinophil counts may be normal or even decreased (9–11). A study of 413 imported cases showed that 23% of confirmed patients had normal eosinophil counts (12); a 2021 meta-analysis also found that approximately one-quarter of patients did not present with eosinophilia at the time of diagnosis (13). In our patient, the absolute eosinophil count was persistently 0.01 × 10^9^/L (0.1%). Clinging to the dogma that “parasitic infection always presents with eosinophilia” would certainly have led to a missed diagnosis. Therefore, in immunocompromised hosts, a normal eosinophil count can never be used to exclude *S. stercoralis* infection.

### 3.4 Rapid pathogen diagnosis: advantages of MetaCAP

The gold standard for diagnosis is larval detection by direct smear, agar culture, concentration methods, or PCR, with sensitivities of 21–100 and 100% specificity (14). Conventional stool microscopy has a missed diagnosis rate as high as 70% due to intermittent larval excretion (15, 16). In our patient, routine stool microscopy was negative for ova and parasites. Therefore, relying exclusively on conventional stool examination would have resulted in delayed treatment. We used BALF MetaCAP, a probe capture-based high-throughput sequencing technology. Compared with conventional metagenomic next-generation sequencing (mNGS), MetaCAP combines host DNA depletion with positive enrichment of microbial sequences, significantly improving sensitivity in low-biomass samples – Zhao et al. demonstrated that MetaCAP was approximately 150-fold more sensitive than mNGS for detecting *B. mandrillaris* in blood samples (1,064 vs. 7 RPM), and successfully detected the pathogen in BALF samples where mNGS yielded no reads (17). In this case, the turnaround time from sample submission to report was only 24 h, identifying *S. stercoralis* (16,030 RPM) and cytomegalovirus (14,397 RPM); all other tested pathogens, including bacteria, fungi, mycobacteria, mycoplasma, chlamydia, rickettsiae, and spirochetes, were negative. As MetaCAP suggested a parasitic infection, we repeated bronchoscopy and performed sputum smear microscopy, which revealed motile *S. stercoralis* larvae, providing a critical basis for targeted therapy.

We did not initially screen for CMV, but MetaCAP unexpectedly detected it alongside *S. stercoralis*, illustrating both the utility and limitations of current CMV diagnostics. CMV serology is simple and cost-effective in immunocompetent hosts, but has limited value in immunocompromised patients due to impaired antibody responses (18). Histopathology is specific for tissue-invasive disease but requires invasive procedures often unsuitable for critically ill patients (18). Quantitative PCR is highly sensitive (82–100%) and is the standard for active CMV infection (18), yet it is hypothesis-dependent and cannot detect pathogens outside its primer panel. By contrast, MetaCAP is a hypothesis-free, broad-spectrum tool that can detect thousands of pathogens simultaneously. In our case, MetaCAP already identified CMV with high reads (14,397 RPM; relative abundance 98.67%), so we did not perform quantitative PCR, as the diagnosis was sufficient to guide therapy. Serology and lung biopsy were also omitted—the former due to limited value in this immunosuppressed host, the latter because of invasiveness in critical illness.

### 3.5 Antiparasitic treatment

For chronic, uncomplicated strongyloidiasis, ivermectin 200 μg/kg as a single or two-day course is the standard treatment (19). However, patients with hyperinfection syndrome have a high parasite burden and an autoinfection cycle, requiring longer treatment or combination therapy. Albendazole (400 mg twice daily for 7 days) is an alternative (20); the combination of ivermectin and albendazole can improve cure rates (21). Our patient weighed 60 kg. He received albendazole 0.4 g twice daily via nasogastric tube combined with ivermectin 12 mg once daily via nasogastric tube. After 1 week, the *S. stercoralis* reads in BALF decreased from 16,030 to 69RPM, and treatment was continued for more than 1 month, ultimately leading to clinical cure. Because the autoinfection cycle of *S. stercoralis* is approximately 2–3 weeks, we recommend that immunocompromised patients receive repeated courses (e.g., every 2–3 weeks) to ensure eradication (22). The patient declined follow-up stool or sputum testing; therefore, we could not justify additional empirical treatment cycles—particularly in the absence of microbiological evidence of persistent infection, given the patient’s good clinical condition, and considering the potential for drug-related adverse effects. As a result, the recommended 2–3 weekly treatment intervals were not continued.

### 3.6 Dilemma and balance in glucocorticoid use

Glucocorticoid use was the most challenging decision. The 2023 American College of Rheumatology (ACR) / American College of Chest Physicians (CHEST) guidelines conditionally recommend glucocorticoids for non-systemic sclerosis systemic autoimmune rheumatic disease-associated interstitial lung disease (SARD-ILD) (23). Our patient had been on maintenance methylprednisolone (4 mg bid) for 8 years and developed severe ARDS after admission. BALF MetaCAP revealed co-infection with *S. stercoralis* and CMV, suggesting infection as the primary driver, though autoimmune flare could not be excluded. The 2024 Society of Critical Care Medicine (SCCM) / American Thoracic Society (ATS) guidelines recommend methylprednisolone 1–2 mg/kg/day for ARDS (24), and conventional practice for connective tissue disease-associated interstitial lung disease (CTD-ILD) exacerbations includes pulse therapy (1 g/day × 3 days) followed by oral prednisolone 1 mg/kg/day (25, 26).

The patient’s ARDS was likely driven by a severe opportunistic infection due to immunosuppression; however, an acute inflammatory response related to autoimmune activity could not be excluded. On the basis of sufficient antimicrobial therapy and after ruling out disseminated infection (the patient was hemodynamically stable, with a Glasgow Coma Scale score of 15 and no significant abnormalities in cardiac, hepatic, or renal function), and in consideration of the above guideline recommendations, we cautiously initiated methylprednisolone at 80 mg every 12 h (patient weight: 60 kg). This dose was lower than the pulse dose typically used for acute exacerbations of connective tissue disease-associated interstitial lung disease, but slightly above the range recommended for ARDS (1–2 mg/kg/day, i.e., 60–120 mg/day for this patient). The dose was then tapered to 8 mg daily over 20 days. This balanced the risks of opportunistic infection and autoimmune activity, aligning with guideline and conventional practice while limiting immunosuppression (24–26). Then the ARDS improved rapidly, parasite burden declined within a few days, and respiratory function returned to baseline during follow-up, suggesting infection was the main cause. This experience supports that short-term glucocorticoids are feasible and safe under rigorous anti-infective coverage and monitoring.

### 3.7 Strengths and limitations

This case has several strengths. First, to our knowledge, it is one of the few reports of severe ARDS caused by *S. stercoralis* hyperinfection in a patient with Sjögren’s syndrome, highlighting an under-recognized complication in this autoimmune population. Second, it demonstrates the diagnostic value of early BALF MetaCAP—particularly when eosinophilia is absent, a potentially misleading feature—enabling rapid pathogen identification and timely targeted therapy. Third, the successful outcome achieved through prompt anti-infective therapy and cautious glucocorticoid use under adequate coverage provides a practical example of balancing infection control and autoimmune disease management in a high-risk host.

Several limitations should also be acknowledged. First, although MetaCAP detected high reads of *S. stercoralis* and CMV, quantitative PCR was not performed to confirm pathogen loads or monitor dynamic changes; CMV serology and lung biopsy were also omitted, limiting comprehensive assessment of active CMV infection. Second, cerebrospinal fluid examination was not performed to exclude intracranial involvement. Third, the glucocorticoid regimen was individualized based on guideline recommendations but lacked a standardized comparator. Fourth, microbiological clearance could not be confirmed after discharge, as the patient declined follow-up testing; however, clinical recovery was sustained at 9 months.

## 4 Conclusion

This case underscores that strongyloidiasis should be suspected in immunocompromised patients with unexplained gastrointestinal or respiratory symptoms, even when eosinophil counts are normal. Early BALF MetaCAP enables rapid pathogen identification and should be considered a first-line diagnostic tool in critically ill patients. Short-term glucocorticoid therapy is reasonable when anti-infective coverage is assured. Furthermore, screening for asymptomatic *S. stercoralis* (via stool microscopy/ELISA) and prophylactic treatment should be considered in high-risk individuals (27).

## Data Availability

The raw data supporting the conclusions of this article will be made available by the authors, without undue reservation.
